# Impact of Prior Intra-articular Injections on the Risk of Prosthetic Joint Infection Following Total Joint Arthroplasty: A Systematic Review and Meta-Analysis

**DOI:** 10.3389/fsurg.2021.737529

**Published:** 2021-09-07

**Authors:** Fei Nie, Wei Li

**Affiliations:** Department of Orthopedics, Chengdu No. 6 People's Hospital, Chengdu, China

**Keywords:** corticosteroids, hyaluronic acid, prosthetic joint infection, joint replacement, osteoarthritis

## Abstract

**Objective:** The current review was designed to assess the impact of prior intra-articular injections on the risk of prosthetic joint infection (PJI) in patients undergoing total joint arthroplasty (TJA) with a focus on the timing of injection before surgery.

**Methods:** The databases of PubMed, Embase and Google Scholar were searched up to 15th June 2021. All studies comparing the incidence of PJI with and without prior intra-articular injections were included. Risk ratios (RR) with 95% confidence intervals were calculated for PJI.

**Results:** Nineteen studies were included. Both corticosteroids and hyaluronic acid injections were used before TJA in the included studies. Overall, comparing 127,163 patients with prior intra-articular injections and 394,104 patients without any injections, we noted a statistically significant increased risk of PJI in the injection group (RR 1.24 95% CI: 1.11, 1.38 I^2^ = 48% *p* = 0.002). On subgroup analysis, there was a statistically significant increased risk of PJI in the injection group in studies where intra-articular injections were administered <12 months before surgery (RR 1.18 95% CI: 1.10, 1.27 I^2^ = 7% *p* < 0.00001). Furthermore, on meta-analysis, we noted non-significant but increased risk of PJI when injections were administered 1 month (RR 1.47 95% CI: 0.88, 2.46 I^2^ = 77% *p* = 0.14), 0–3 months (RR 1.22 95% CI: 0.96, 1.56 I^2^ = 84% *p* = 0.11), and 3–6 months (RR 1.16 95% CI: 0.99, 1.35 I^2^ = 49% *p* = 0.06) before surgery.

**Conclusion:** Our results indicate that patients with prior intra-articular injections have a small but statistically significant increased risk of PJI after TJA. Considering that PJI is a catastrophic complication with huge financial burden, morbidity and mortality; the clinical significance of this small risk cannot be dismissed. The question of the timing of injections and the risk of PJI still remains and can have a significant impact on the decision making.

**Systematic Review Registration:** PROSPERO: CRD42021258297.

## Introduction

Osteoarthritis (OA) of the knee and hip joints is a debilitating condition that has a high prevalence worldwide. While the disease is not fatal, it has a significant impact on joint function resulting in pain and reduced range of motion (1). In the early stages of OA, conservative treatment modalities like activity modification, physical therapy, exercise are recommended to delay disease progression, however, they are often not effective in providing rapid relief in patients with symptomatic OA (2).

In patients with symptomatic and end-stage OA, intra-articular injections are frequently administered for both diagnostic and therapeutic purposes (3–5). Such injections are helpful to delineate the cause of pain arising from the intra-articular source or due to extra-articular causes like the spine or surrounding musculature (6). Furthermore, several high-quality randomized controlled trials (RCTs) have demonstrated the efficacy of intra-articular injections of corticosteroids (CS) or hyaluronic acid (HA) in managing OA of the knee and hip joints (3, 7). These injections can significantly alleviate pain and improve symptoms in short term thereby delaying the need for total joint arthroplasty (TJA). Indeed, guidelines of the American College of Rheumatology conditionally advocate the use of CS injections for managing OA while the Osteoarthritis Research Society International suggests the use of intra-articular CS for symptomatic OA not responsive to anti-inflammatory drugs and those of signs of local inflammation (8, 9). Despite the results from several RCTs, the use of intra-articular injections has been controversial as well. The use of intra-articular drugs, especially CS, has been associated with rapid progression of osteoarthritis, subchondral insufficiency fracture, osteonecrosis, and rapid joint destruction with bone loss (10). Several other local complications like skin and fat atrophy, septic arthritis, and prosthetic joint infections (PJI) have also been related to the use of intra-articular injections (5, 11).

In the past decade, several systematic reviews and meta-analyses have dwelled on the relationship between the use of prior intra-articular injections and the risk of PJI after TJA (11–15). However, these reviews had several limitations. Foremost, the number of studies included was not high with a maximum of eight comparative studies pooled in the meta-analysis. Furthermore, none of the past reviews took into account the timing of injection and the risk of PJI. Given these shortcomings, there is a need for an updated and comprehensive review on this subject. Therefore, the current review was designed to assess the impact of prior intra-articular injections on the risk of PJI in patients undergoing TJA with a focus on the timing of injection before surgery.

## Materials and Methods

### Research Question

The research question of interest was: Do injections of intra-articular drugs before TJA increase the risk of PJI? The methodology of this review was based on the guidelines of the PRISMA statement (Preferred Reporting Items for Systematic Reviews and Meta-analyses) (16). The review protocol was registered on PROSPERO (no CRD42021258297).

### Literature Search

The databases of PubMed, Embase and Google Scholar were searched by two reviewers independent of each other. All databases were searched from their inception to 15th June 2021. The keywords used were: “arthroplasty”, “joint replacement”, “injections”, “infection”, “corticosteroids”, and “hyaluronic acid”. Supplementary Table 1 presents details of the literature search. After deduplication of the search results, we reviewed the output of each database by assessing the titles and abstracts of every study. We identified articles relevant to the review and extracted their full texts. The two reviewers independently evaluated these studies for final inclusion in the review. We resolved any disagreements by discussion. In the end, we reviewed the reference list of included studies for any missed references.

### Eligibility Criteria

The PICOS (Population, Intervention, Comparison, Outcome, Study type) inclusion criteria of the review were as follows:-

Population: Adult patients undergoing TJA [total hip arthroplasty (THA) or total knee arthroplasty (TKA)].
Intervention: intra-articular injections of any drug before TJA.
Comparison: no injections before TJA.
Outcome: PJI.
Study type: all prospective or retrospective cohort studies, RCTs, and clinical controlled trials.

We did not pre-define PJI and used the definition from the included studies.

We excluded the following studies: (1) Studies comparing infected and non-infected cases of TJA (2) Studies not reporting incidence of PJI (3) Studies not comparing outcomes with a control group (4) Non-English language studies, abstracts, case reports, and review articles. (5) Studies reporting duplicate data. In case there were multiple studies from the same healthcare setup or database, we included the study with the largest sample size.

### Data Extraction and Quality Assessment

Data from each study was sourced by two authors independently. We extracted details of the first author, publication year, study type, study location, the database used, joint studied, sample size, mean age, a drug injected, time from injection to surgery, the definition of PJI, duration of follow-up, and study outcomes.

The methodological quality of included studies was assessed using the Newcastle-Ottawa scale (NOS) (17). This too was carried out in duplicate and independently by two study investigators. Studies were awarded points for selection of study population, comparability, and outcomes. The maximum score which can be awarded is nine.

### Statistical Analysis

We conducted the meta-analysis using “Review Manager” (RevMan, version 5.3; Nordic Cochrane Centre [Cochrane Collaboration], Copenhagen, Denmark; 2014). A random-effects model was preferred. We pooled the incidence of PJI using risk ratios (RR) with 95% confidence intervals (CI). A sensitivity analysis was also performed by sequentially excluding individual studies to check any undue influence of the study on the total effect size. A sub-group was performed based on the timing of injection before TJA. We also conducted further subgroup analyses based on the joint type and drug injected where possible. Heterogeneity was assessed using the I^2^ statistic. I^2^ values of 25–50% represented low, values of 50–75% medium, and more than 75% represented substantial heterogeneity. We used funnel plots to assess publication bias.

## Results

### Search and Study Details

**Figure 1:**
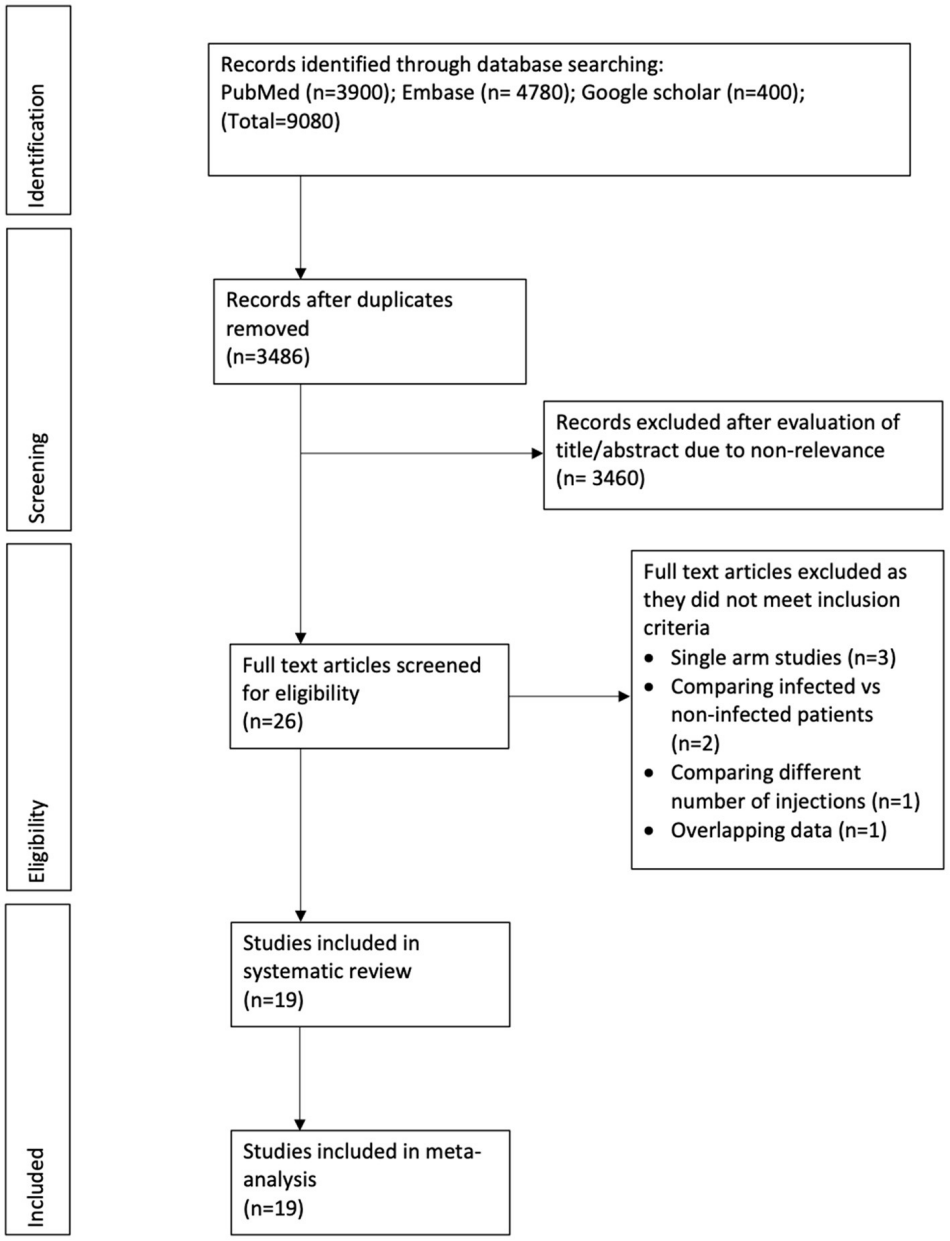
Study flow chart.

The study flow chart is presented in Figure 1. After a detailed literature search and deduplication of results, a total of 3,486 unique articles were assessed. Based on the title and abstract screening, 3,460 articles were excluded and full texts of 26 studies were evaluated for inclusion in the review. Seven studies were excluded with reasons. Finally, 19 studies were included in this systematic review and meta-analysis (18–36).

Characteristics of included studies are presented in Table 1. The included studies were published between 2005 to 2021. Six studies were conducted in Europe while the remaining in North America. Except for one, all were retrospective cohort studies. Nine studies carried out baseline batching of study and control groups. Ten studies were on the hip joint, eight on the knee, and one on both hip and knee. Nine studies assessed the effect of CS injections, one was on only HA injections, while the remaining included a sample of both CS and HA injections. Three studies included only those patients who had received injections <6 months before surgery while 10 studies assessed the impact of intra-articular injections administered <12 months before surgery. The definition of PJI was not universally reported and was variable across studies. The follow-up amongst included studies ranged from three months to a maximum of seven years. The NOS score of the studies ranged from 5–8.

**Table 1:** Details of included studies.

**Study**	**Location**	**Database**	**Study type**	**Joint**	**Drug injected**	**Sample size**		**Mean age**		**Time from injection to surgery**	**Criteria for PJI**	**Follow-up**	**NOS score**
						**Study**	**Control**	**Study**	**Control**				
Tang et al. (19)	USA	NYU Langone Health	R	Knee & Hip	CS or HA	342	2,998	65.5	65	Hip: 12.4± 11 monthsKnee: 20± 17.4 months	NR	3 months	7
Grondin et al. (29)	France	University Hospital of Nantes or other clinics	P	Knee	CS or HA	207	97	NR	NR	< 6 months	International Consensus Meeting on PJI (at least one of the two major criteria: Two positive growths of the same organism using standard culture methods, or sinus tract with evidence of communication to the joint or visualization of the prosthesis)	24.9± 3.8 months	5
Forlenza et al. (30)	USA	PearlDiver database (2011-2018)	R^*^	Hip	CS	29,058	29,058	NR	NR	< 6 months	NR	6 months	8
Kurtz et al. (31)	USA	Medicare (2010-2017)	R	Knee	CS or HA	33,331	56,506	NR	NR	< 12 months	Infections treated by revision, arthrotomy or spacer insertion	2 years	8
Colen et al. (32)	Netherland	University of Amsterdam	R	Hip	HA	118	495	69.1	68.2	< 6 months	Definition from the workgroup of the Musculoskeletal Infection Society	52 months	6
Bedard et al. (33)	USA	Humana database (2007-2014)	R	Knee	CS or HA	29,603	54,081	NR	NR	< 12 months	Infections requiring surgical intervention	6 months	6
Werner et al. (18)	USA	Medicare (2005-2011)	R	Hip	CS or HA	3,368	31,229	NR	NR	< 12 months	NR	6 months	6
Schairer et al. (34)	USA	State-level ambulatory surgery and inpatient databases for Florida and California	R	Hip	CS or HA	5,421	16,8537	66.8	66.6	< 12 months	Infections requiring surgical intervention	1 year	8
Khanuja et al. (23)	USA	Johns Hopkins University	R^*^	Knee	CS	302	302	66	65	< 12 months	Centers for Disease Control and Prevention/ National Healthcare Safety Network definitions	3.5 years	8
Amin et al. (22)	USA	Loma Linda University	R	Knee	CS or HA	783	845	NR	NR	< 12 months	Definition from the workgroup of the Musculoskeletal Infection Society	Up to 7 years	6
Ravi et al. (21)	Canada	Ontario Health Insurance Plan	R	Hip	CS or HA	1,691	35,413	67	68	< 12 months	NR	2 years	8
Cancienne et al. (36)	USA	Medicare (2005-2011)	R^*^	Knee	CS or HA	22,240	13,650	NR	NR	< 12 months	NR	6 months	8
Croft et al. (20)	Canada	Memorial University of Newfoundland	R^*^	Hip	CS	48	48	61.5	62.7	Mean 5.9 months	Infections requiring surgical intervention	10.45 months	7
Meermans et al. (35)	Belgium	Lievensberg Hospital & University Hospital Pellenberg	R^*^	Hip	CS	175	175	66.4	66.6	< 12 months	Defined as (1) a sinus tract communicating with the implant, (2) the identical pathogen isolated from two or more separate tissue samples, or (3) the presence of purulence in the joint.	71 months	8
Desai et al. (24)	UK	Wrightington Hospital,	R^*^	Knee	CS	90	180	NR	NR	45 cases within 12 months	Defined as positive swab cultures or deep tissue biopsy, exploration/washout of wound with positive culture, revision surgery for infection	1 year	7
Sreekumar et al. (25)	UK	Wrightington Hospital,	R^*^	Hip	CS	68	136	NR	NR	Median 11 months	NR	1 year	7
Papavasiliou et al. (26)	UK	Eastbourne district general hospital	R	Knee	CS	54	90	NR	NR	< 11 months	Occurring within 1 year of surgery, and met at least one of the following: 1. Purulent drainage, 2. culture from aseptically aspirated fluid, swab/tissue biopsy from the deep-tissue layers or pus cells on microscopy 3. deep incision which spontaneously dehisced or was explored for a temperature of >38 °C, localized pain or tenderness 4. an abscess or other evidence of infection involving the deep incision 5. diagnosis of a deep infection by clinician.	1 year	6
Mcintosh et al. (27)	USA	Mayo clinic	R^*^	Hip	CS	224	224	70	69	Mean 112 days	NR	NR	6
Kaspar et al. (37)	Canada	McMaster university	R^*^	Hip	CS	40	40	71	70.5	0.5-42.9 months	Infections requiring revision	29.8 months	8

*R, retrospective; P, prospective; NR, not reported; CS, corticosteroid; HA, Hyaluronic acid; PJI, prosthetic joint infection; NOS, Newcastle Ottawa scale*.

* *Matched cohort*.

### Meta-Analysis

**Figure 2:**
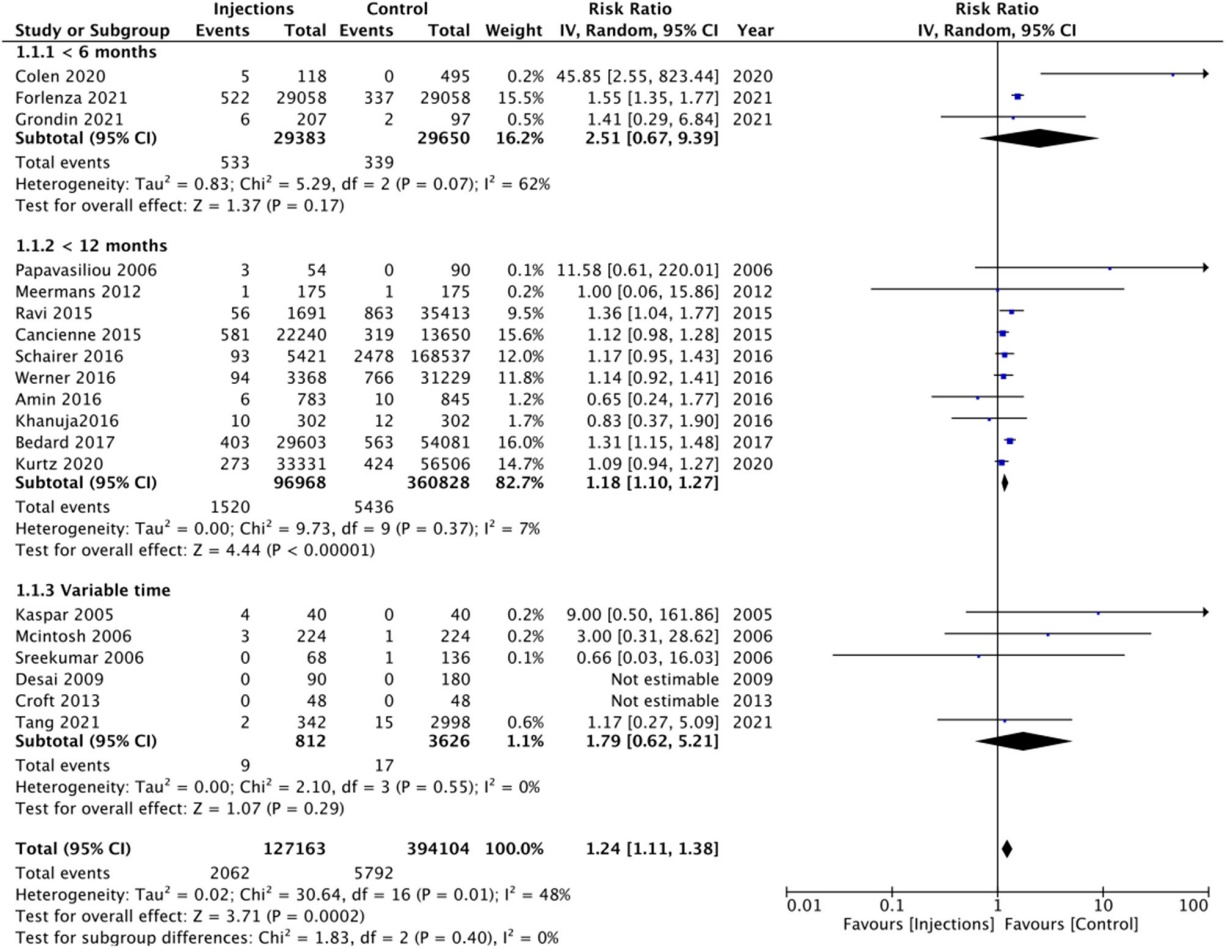
Meta-analysis of all studies reporting PJI between injection and control groups with sub-group analysis based on timing of injection before surgery.

On pooled analysis of all 19 studies, comparing 127,163 patients with prior intra-articular injections and 394,104 patients without any injections, we noted a statistically significant increased risk of PJI in the injection group (RR 1.24 95% CI: 1.11, 1.38 I^2^ = 48% *p* = 0.002) (Figure 2). However, since the included studies varied significantly in the time of injection before surgery, we explored the inter-study heterogeneity using a sub-group analysis. We noted a statistically significant increased risk of PJI in the injection group in studies where intra-articular injections were administered <12 months before surgery (RR 1.18 95% CI: 1.10, 1.27 I^2^ = 7% *p* < 0.00001). However, no such difference was seen on a pooled analysis of the three studies including only those patients who had received injections <6 months before surgery (RR 2.51 95% CI: 0.67, 9.39 I^2^ = 62% *p* = 0.17) (Figure 2). Overall, there was no evidence of publication bias (Supplementary Figure 1). On sensitivity analysis, there was no change in the significance of the overall results with the exclusion of any study.

We conducted a further subgroup analysis of the 10 studies with <12 months' time interval between injection and surgery (Table 2). The type of joint (hip or knee) did not have any impact on the results as a statistically significant increase in the risk of PJI was noted with both. Amin et al. and Kurtz et al. also reported separate data for the risk of PJI with CS injections. On subgroup analysis of five studies assessing only CS administration <12 months before surgery, we noted no significant increase in the risk of PJI (Table 2).

**Table 2:** Subgroup analysis of PJI for studies reporting time interval between intra-articular injections and surgery.

**Variable**	**Number studies**	**Sample size of injection group**	**Sample size of control group**	**Risk ratio**
**<12 months**				
Hip joint	4	10,655	235,354	1.20 95% CI: 1.05, 1.36 I^2^ = 0% *p* = 0.006
Knee joint	6	86,313	125,474	1.16 95% CI: 1.02, 1.32 I^2^ = 41% *p* = 0.02
Corticosteroid only	5	21,634	57,918	1.00 95% CI: 0.84, 1.98 I^2^ = 0% *p* = 0.99
**0–3 months**				
Hip joint	3	16,266	228,824	1.37 95% CI: 1.19, 1.57 I^2^ = 0% *p* < 0.00001
Knee joint	5	30,009	102,209	1.08 95% CI: 0.70, 1.66 I^2^ = 90% *p* = 0.73
**3–6 months**				
Hip joint	3	16,516	228,824	1.11 95% CI: 0.86, 1.43 I^2^ = 56% *p* = 0.44
Knee joint	4	19,216	68,878	1.18 95% CI: 0.91, 1.54 I^2^ = 59% *p* = 0.22

**Figure 3:**
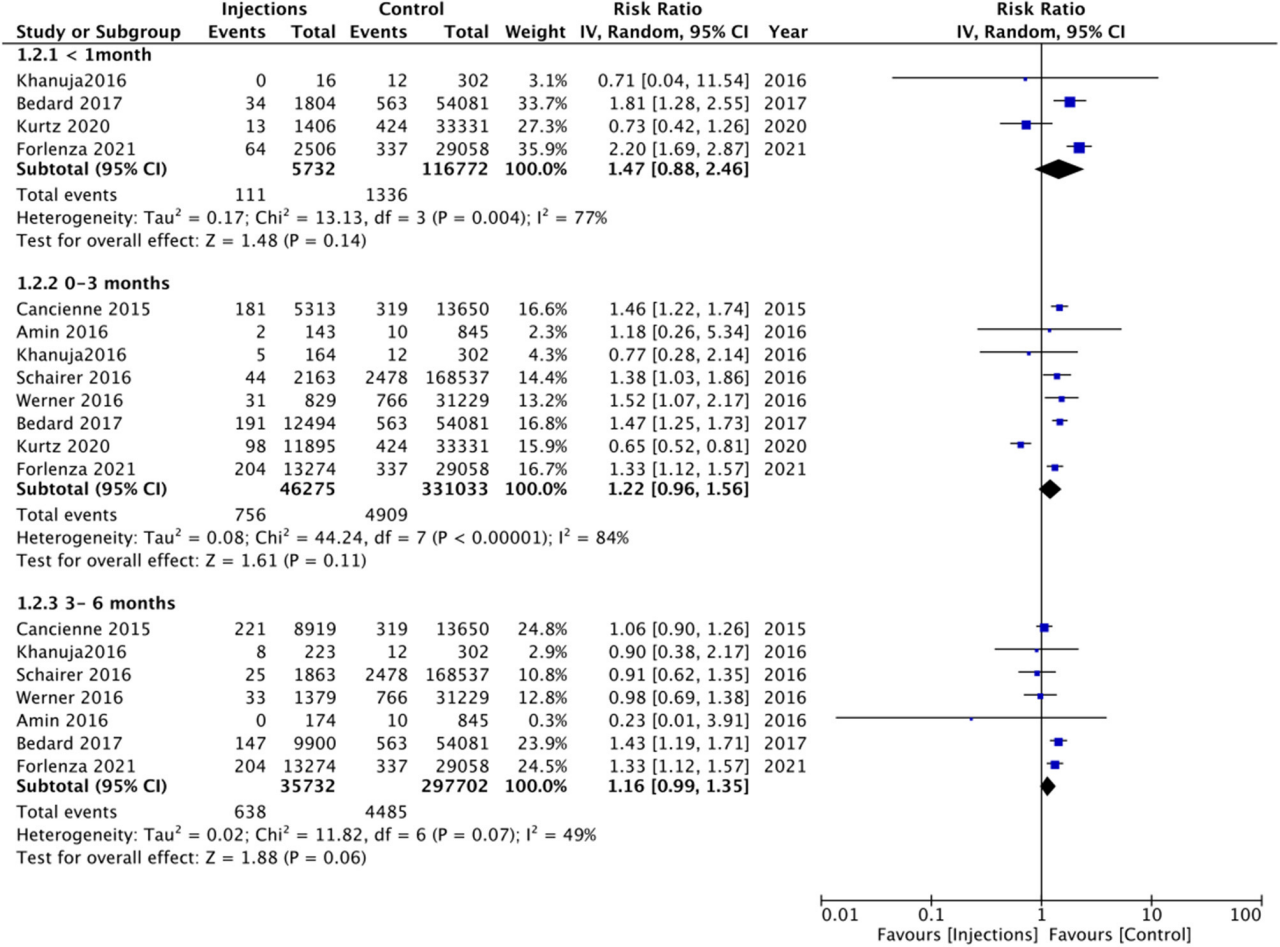
Meta-analysis of PJI between injection and control groups with sub-group analysis based on timing of injection before surgery as <1 months, 0–3 months, and 3–6 months.

In addition to the <6 months and <12 months' time intervals, several studies also reported the risk of PJI with injections given 1 month, 0–3 months, and 3–6 months before surgery. On meta-analysis, we noted no significant increased risk of PJI when injections were administered 1 month (RR 1.47 95% CI: 0.88, 2.46 I^2^ = 77% *p* = 0.14), 0–3 months (RR 1.22 95% CI: 0.96, 1.56 I^2^ = 84% *p* = 0.11), and 3–6 months (RR 1.16 95% CI: 0.99, 1.35 I^2^ = 49% *p* = 0.06) before surgery (Figure 3). On further analysis of studies included in the 0–3 month subgroup, we noted a significantly higher risk of PJI for studies on the hip joint but not on the knee joint (Table 2). However, no such difference was noted on further analysis of studies in the 3–6 months subgroup (Table 2).

## Discussion

PJI is a debilitating complication seen in 1–2% of patients undergoing TJA and accounts for around 15% of total revision procedures (37). Notwithstanding the intense research in the prevention and management of PJI, there seems to be no decline in the incidence of this devastating complication (38, 39). Prediction models indicate that the incidence of PJI for knee and hip joints is on the rise and is expected to grow by 170 and 176% respectively (39). Amongst the several risk factors identified for PJI, the role of prior intra-articular injections has been controversial. Despite several studies and systematic reviews, it is still unclear how do prior CS and HA injections impact the incidence of PJI (11–15).

The previous meta-analysis on this subject with a small number of studies has produced contrasting results. Charalambous et al. (15) in a pooled analysis of eight studies reported no significant increase in the risk of PJI with prior intra-articular CS injections (RR 1.87; 95% CI 0.80–4.35). On the other hand, Xing et al. (14) demonstrated a significantly higher risk of PJI in patients with prior history of intra-articular CS injections [Odds ratio (OR) 2.13, 95% CI 1.02–4.45]. Li et al. (13) in a review of systematic reviews pointed out the scarcity of evidence and the need for further studies to clarify the role of prior injections on the risk of PJI. In this context, our review is a significant update of prior studies by providing cumulative evidence from 19 studies with 521,267 participants. Our results demonstrated that patients with prior history of intra-articular injections have a statistically significant 24% increased risk of PJI. Overall, the risk of PJI in the injection group was 1.6% while in the control group it was 1.47%. While these figures are within the range reported in the literature (37), the significant increased incidence of PJI with prior intra-articular injections cannot be dismissed. PJI is a catastrophic complication with huge financial burden, morbidity and mortality (38, 39); and the small increased risk needs to be interpreted in the magnitude of the potentially preventable complication.

Recently, it has been suggested that the timing of injection before surgery is an important variable while assessing the risk of PJI (30). Indeed, since the pathophysiological mechanism for the heightened risk of infection with prior injections is based on the prolonged immunosuppressive effects of CS and the joint contamination caused by the injection procedure (15), it would be plausible to believe that reduced time interval between injection and surgery would increase the risk of PJI. To explore such a relationship, we performed multiple subgroup analyses based on the timing of injections but with contrasting results. In the largest subgroup of studies with <12 months' time interval between injections and surgery, we noted a statistically significant 10% increased risk of PJI in the injection group. However, on a meta-analysis of studies with a time interval of <6 months, 3–6 months, 0–3 months, and <1month between injection and surgery, we noted no significant increase in the risk of PJI. Nevertheless, on careful examination of the effect size and 95% CIs, it can be noted that there was a tendency of increased risk of PJI in the injection cohort in all these subgroups but the difference could not reach statistical significance. One reason for this could be the limited number of studies in each analysis which reduced the statistical power. Since the risk of PJI is only marginally increased with prior intra-articular injections, the number of participants in the injection cohort of these subgroups may not have been sufficient to produce statistically significant results.

The majority of studies in our review did not present separate data for CS and HA which limited our ability to differentiate the risk of PJI with these two drugs. In a small subgroup analysis of five studies with <12 months' time interval between injections and surgery, we noted no significant impact of only CS injections on the risk of PJI. To date, very few studies have compared the risk of PJI with prior HA and CS. Kurtz et al. (31) in a large retrospective review have noted no difference in the risk of PJI with prior CS or HA injections. Similarly, Amin et al. (22) also found no difference between the two drugs while assessing the risk of PJI with prior intra-articular injections. Given the scarcity of data, further studies comparing the two drugs for the risk of subsequent PJI are needed to derive stronger conclusions.

Another factor not considered in our results is the number of injections before surgery. However, a few studies have examined this issue. In one of the excluded studies, Richardson et al. (40) compared the risk of PJI after TKA between those receiving multiple injections vs. those receiving single intra-articular injections before surgery and found no difference in the risk of PJI. On the other hand, Forlenza et al. (30) have demonstrated a dose-dependent relationship between the number of injections and risk of PJI after THA with every unit increase in the number of injections increasing the risk of infections. These contrasting results between THA and TKA are difficult to explain especially when our meta-analysis has failed to demonstrate significant differences in complication rates between THA and TKA. In another study, Kokubun et al. (41) have compared infection rates in TKA patients receiving ≤ 3 vs. >4 injections before surgery and found no difference between the two groups. Given such heterogeneous results, the exact relationship between the number of injections and the risk of PJI too deserves further research.

The increased risk of PJI with prior intra-articular injections seen in our meta-analysis should be interpreted with the fact that several confounders can influence this outcome. Research has shown that comorbidities like diabetes, obesity, malnutrition, smoking, tobacco use, narcotic use, alcohol dependence, and methicillin-resistant *Staphylococcus aureus* colonization of the nares can increase the risk of PJI (42). Tang et al. (19) in their study assessing predictive factors for use of intra-articular injections before TJA have demonstrated that less healthy patients who eventually require TJA more often undergo intra-articular injections to delay surgery as compared to healthier patients. Therefore, it is plausible that baseline differences between the injection and control groups could contribute to the difference In the risk of infection. To nullify this effect, baseline matching and multivariate analysis are essential. While matched cohorts were used in a few studies, multivariable-adjusted data were not reported by majority studies, prohibiting a pooled analysis. In the few studies reporting multivariable-adjusted results, some demonstrated no increased risk of PJI (19, 31) while others demonstrated an increased risk (21, 34).

The limitations of our review need to be mentioned. Foremost, our analysis is based on data mostly from retrospective cohort studies which have an inherent bias. There would have been obvious selection bias between the injection and control groups which could have skewed the results. Secondly, the majority of our studies were from administrative databases, and data was collected using current procedural terminology (CPT) codes. It is known that such databases are prone to coding errors. Furthermore, since the majority of studies were from databases in the USA, despite taking care to avoid overlapping studies, we may have inadvertently repeated the same patients. Thirdly, all of the studies were from North America and Europe and this limits the generalizability of our results to the global population. Fourthly, due to a lack of data, we were unable to assess the impact of the number of injections and type of drug (CS or HA) on the study outcomes. Due to the same reason, we were unable to pool multivariable-adjusted ratios for the risk of PJI. Lastly, the definition of PJI was either not reported or were varied in the included studies. This has important implications as lack of standardized definition may have overestimated or underestimated the incidence of PJI in the included studies. Also, there was heterogeneity in the duration of follow-up ranging from just 3 months to up to 7 years.

To conclude, the results of our updated systematic review and meta-analysis indicate that patients with prior intra-articular injections have a small but statistically significant increased risk of PJI after TJA. Considering that PJI is a catastrophic complication with huge financial burden, morbidity and mortality; the clinical significance of this small risk cannot be dismissed. The question of the timing of injections and the risk of PJI still remains and can have a significant impact on the decision making. Further high-quality data is needed focussing especially on the timing, type and number of injections and the risk of PJI after TJA. Future studies should also use standardized definitions of PJI to allow comparisons between different cohorts.

## Data Availability Statement

The original contributions presented in the study are included in the article/Supplementary Material, further inquiries can be directed to the corresponding author/s.

## Author Contributions

FN and WL conceived and designed the study. FN was involved in literature search and data collection and wrote the paper. WL analyzed the data and reviewed and edited the manuscript. All authors read and approved the final manuscript.

## Conflict of Interest

The authors declare that the research was conducted in the absence of any commercial or financial relationships that could be construed as a potential conflict of interest.

## Publisher's Note

All claims expressed in this article are solely those of the authors and do not necessarily represent those of their affiliated organizations, or those of the publisher, the editors and the reviewers. Any product that may be evaluated in this article, or claim that may be made by its manufacturer, is not guaranteed or endorsed by the publisher.
