# Supplementary material for: Impact of Prior Intra-articular Injections on the Risk of Prosthetic Joint Infection Following Total Joint Arthroplasty: A Systematic Review and Meta-Analysis
Source: Front Surg. 2021 Sep 7;8:737529. doi: 10.3389/fsurg.2021.737529 (PMC8452968; doi:10.3389/fsurg.2021.737529)
Supplement: Supplementary file 1 [file Table_1.DOCX]

Supplementary Table 1: Search strategy

| **Query** | **Search Details** |
| --- | --- |
| ((arthroplasty) AND (injection)) AND (infection) | ("arthroplasty"[MeSH Terms] OR "arthroplasty"[All Fields] OR "arthroplasties"[All Fields]) AND ("inject"[All Fields] OR "injectability"[All Fields] OR "injectant"[All Fields] OR "injectants"[All Fields] OR "injectate"[All Fields] OR "injectates"[All Fields] OR "injected"[All Fields] OR "injectible"[All Fields] OR "injectibles"[All Fields] OR "injecting"[All Fields] OR "injections"[MeSH Terms] OR "injections"[All Fields] OR "injectable"[All Fields] OR "injectables"[All Fields] OR "injection"[All Fields] OR "injects"[All Fields]) AND ("infect"[All Fields] OR "infectability"[All Fields] OR "infectable"[All Fields] OR "infectant"[All Fields] OR "infectants"[All Fields] OR "infected"[All Fields] OR "infecteds"[All Fields] OR "infectibility"[All Fields] OR "infectible"[All Fields] OR "infecting"[All Fields] OR "infection s"[All Fields] OR "infections"[MeSH Terms] OR "infections"[All Fields] OR "infection"[All Fields] OR "infective"[All Fields] OR "infectiveness"[All Fields] OR "infectives"[All Fields] OR "infectivities"[All Fields] OR "infects"[All Fields] OR "pathogenicity"[MeSH Subheading] OR "pathogenicity"[All Fields] OR "infectivity"[All Fields]) |
| (((corticosteroids)) OR (hyaluronic acid)) AND (joint replacement) | ("adrenal cortex hormones"[MeSH Terms] OR ("adrenal"[All Fields] AND "cortex"[All Fields] AND "hormones"[All Fields]) OR "adrenal cortex hormones"[All Fields] OR "corticosteroid"[All Fields] OR "corticosteroids"[All Fields] OR "corticosteroidal"[All Fields] OR "corticosteroide"[All Fields] OR "corticosteroides"[All Fields] OR ("hyaluronic acid"[MeSH Terms] OR ("hyaluronic"[All Fields] AND "acid"[All Fields]) OR "hyaluronic acid"[All Fields])) AND ("arthroplasty, replacement"[MeSH Terms] OR ("arthroplasty"[All Fields] AND "replacement"[All Fields]) OR "replacement arthroplasty"[All Fields] OR ("joint"[All Fields] AND "replacement"[All Fields]) OR "joint replacement"[All Fields]) |
| (((corticosteroids)) OR (hyaluronic acid)) AND (arthroplasty) | ("adrenal cortex hormones"[MeSH Terms] OR ("adrenal"[All Fields] AND "cortex"[All Fields] AND "hormones"[All Fields]) OR "adrenal cortex hormones"[All Fields] OR "corticosteroid"[All Fields] OR "corticosteroids"[All Fields] OR "corticosteroidal"[All Fields] OR "corticosteroide"[All Fields] OR "corticosteroides"[All Fields] OR ("hyaluronic acid"[MeSH Terms] OR ("hyaluronic"[All Fields] AND "acid"[All Fields]) OR "hyaluronic acid"[All Fields])) AND ("arthroplasty"[MeSH Terms] OR "arthroplasty"[All Fields] OR "arthroplasties"[All Fields]) |
| (joint replacement) AND (injection) | ("arthroplasty, replacement"[MeSH Terms] OR ("arthroplasty"[All Fields] AND "replacement"[All Fields]) OR "replacement arthroplasty"[All Fields] OR ("joint"[All Fields] AND "replacement"[All Fields]) OR "joint replacement"[All Fields]) AND ("inject"[All Fields] OR "injectability"[All Fields] OR "injectant"[All Fields] OR "injectants"[All Fields] OR "injectate"[All Fields] OR "injectates"[All Fields] OR "injected"[All Fields] OR "injectible"[All Fields] OR "injectibles"[All Fields] OR "injecting"[All Fields] OR "injections"[MeSH Terms] OR "injections"[All Fields] OR "injectable"[All Fields] OR "injectables"[All Fields] OR "injection"[All Fields] OR "injects"[All Fields]) |
