# Supplementary figures and images for: Impact of Prior Intra-articular Injections on the Risk of Prosthetic Joint Infection Following Total Joint Arthroplasty: A Systematic Review and Meta-Analysis
Source: Front Surg. 2021 Sep 7;8:737529. doi: 10.3389/fsurg.2021.737529 (PMC8452968; doi:10.3389/fsurg.2021.737529)

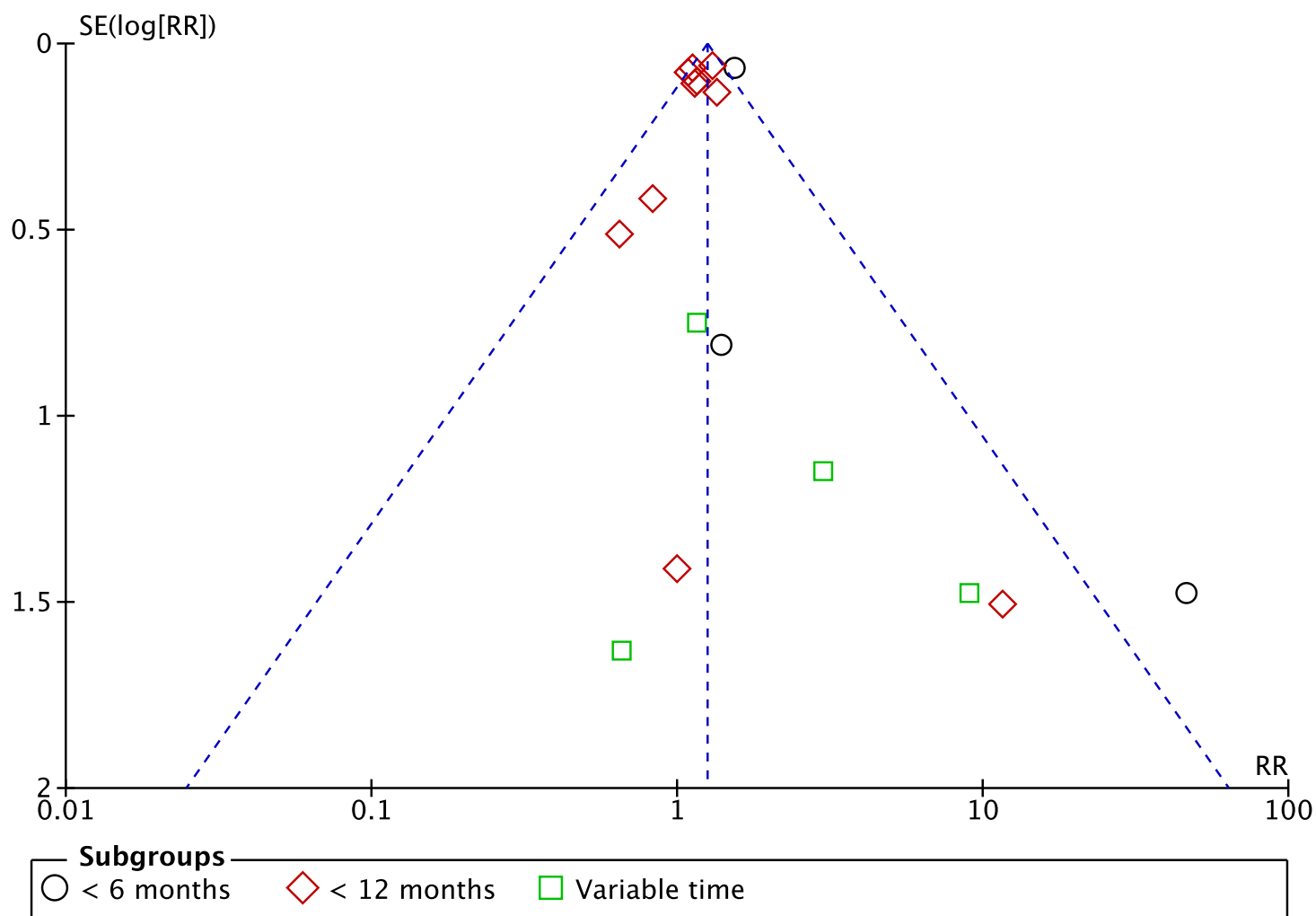

Supplement: Supplementary file 2 [file Data_Sheet_1.PDF]
